# Piezoelectric Bimorphs' Characteristics as In-Socket Sensors for Transfemoral Amputees

**DOI:** 10.3390/s141223724

**Published:** 2014-12-10

**Authors:** Amr M. El-Sayed, Nur Azah Hamzaid, Noor Azuan Abu Osman

**Affiliations:** 1 Department of Biomedical Engineering, Faculty of Engineering, University of Malaya, Kuala Lumpur 50603, Malaysia; E-Mails: azah.hamzaid@um.edu.my (N.A.H), azuan@um.edu.my (N.A.A.O.); 2 Mechatronics Section, Mechanical Engineering Department, Faculty of Engineering, Assiut University, Assiut 71516, Egypt

**Keywords:** in-socket sensor, piezoelectric bimorph, stump/socket pressure

## Abstract

Alternative sensory systems for the development of prosthetic knees are being increasingly highlighted nowadays, due to the rapid advancements in the field of lower limb prosthetics. This study presents the use of piezoelectric bimorphs as in-socket sensors for transfemoral amputees. An Instron machine was used in the calibration procedure and the corresponding output data were further analyzed to determine the static and dynamic characteristics of the piezoelectric bimorph. The piezoelectric bimorph showed appropriate static operating range, repeatability, hysteresis, and frequency response for application in lower prosthesis, with a force range of 0–100 N. To further validate this finding, an experiment was conducted with a single transfemoral amputee subject to measure the stump/socket pressure using the piezoelectric bimorph embedded inside the socket. The results showed that a maximum interface pressure of about 27 kPa occurred at the anterior proximal site compared to the anterior distal and posterior sites, consistent with values published in other studies. This paper highlighted the capacity of piezoelectric bimorphs to perform as in-socket sensors for transfemoral amputees. However, further experiments are recommended to be conducted with different amputees with different socket types.

## 1. Introduction

Advancements in prosthetic knee systems are of increasing importance to assist transfemoral amputees perform their different daily activities [1] such as walking, stair climbing, and running [2,3] more naturally. Prosthetic knee devices are categorized into passive and active types [4]. In order to assist the amputees to replicate such daily movements, active knee devices have to be used to perform those functions. Active knee systems imply that the amputee can interact with the device to facilitate his/her movements. In other words, improving the sensory system of the active knee device shall assist amputees to perform their activities better and more efficiently. Therefore, the development of a prosthetic knee control system is related to sensory signals which facilitate the design of the control algorithm [3,5]. Different types of sensors are involved in active knee devices, for example, a potentiometer acts as an angle sensor to measure the knee joint angle, a load cell is used to measure the knee torque, a gyroscope sensor to detect the acceleration of the knee joint, and a force sensing resistor (FSR) is utilized as on/off sensor to detect the prosthetic knee phases [6]. Each sensor measures a certain parameter. For example the angle sensor (potentiometer) measures the inclination angle of the knee joint during the stride, while a torque sensor identifies the amount of torque that is needed for the knee to perform the movement [7]. These sensors are called passive sensors [6,8,9], as they are placed around the prosthetic knee joint to identify the knee movement. Nevertheless, the interaction between the socket and the amputee subjects is not involved in identifying the knee movement. The direct contact between the amputee subject and the socket device in the presence of the in-socket sensor would be more useful to acquire direct measurements from specific socket locations.

So far, to receive input signal from the stump muscles, electromyography systems (EMGs) were used to detect the muscle activities. An EMG embedded in an active knee system reads the interaction from the user as they detect the user's flexor and extensor muscle activities, generally from the rectus femoris, vastus lateralis, vastus medialis, biceps femoris, and semitendinosus. In order to make use of the EMG signals, such signals are analyzed to formulate the control algorithm that assists the amputee to control the torque in activities such as stair ascent/descent. However, EMG signals measure the muscle activity without considering the reaction forces and moments generated from the ground via the socket by means of pressure distribution. Considering the measurement of the pressure distribution inside the socket that originated from the ground reaction forces to understand the stress distribution during stride might be useful for gait phase identification.

Unlike measuring static pressure distribution such as the interface pressure on the buttocks, the pressure characteristics between the prosthetic socket wall and the stump would have a dynamic interaction between the socket interface pressure [10]. One example of transducer is the load cell, which has different types such as strain gauges to detect force in various applications [11]. Strain gauges are also being used in applications such as wind-tunnel balances and force sensors for robot linkages [12]. Measurement of the interactive forces between human hand and limb rehabilitation devices is achieved using a custom four degree of freedom strain gauge [13]. However, strain gauges show better behavior for static force measurements rather than dynamic investigations, as they show some limitations in the transient responses compared to piezoelectric (PVDF) materials [14]. Another sensing element that is appropriate for detection of dynamic measurements is the piezoelectric material. A piezoelectric bimorph is considered an active element, thus no external power is required to activate the sensor [15]. Moreover, one advantages of the piezoelectric bimorph is that it can adapt to vibrations in such dynamic applications. Piezoelectric bimorphs are among the most widely used sensors in academic research and industrial applications [16].

Piezoelectric materials with a bimorph configuration are used as sensors/actuators in many fields including industrial, aerospace, and medical systems [17–19]. Upon applying a load to the surface of the bimorph, an electrical charge is produced. The relation between the applied force *versus* the piezoelectric bimorph and the output deflection is essential in surgical applications and micro-gripping of fragile objects [16,19]. The charge generated inside the bimorph is measurable in volts, which is proportional to the load applied across its surface [16]. Because of the ability of the piezoelectric bimorph to be used as both the sensor/actuator element [19], current research aims to leverage the advantage of using the piezoelectric bimorph as a sensing element to detect the distribution of the pressure in transfemoral amputees' stump/sockets. In addition, the approach presented in this paper should provide better understanding of the gait characteristics of transfemoral amputees and assist the fabrication process of various socket types [20]. The appropriate location of the sensor inside the socket's wall would provide flexibility to the amputee while wearing the socket and improve the interaction during different activities. On the other hand, researchers in the lower prosthesis field are searching for alternative techniques to improve the sensory system of prosthetic knee devices. Such techniques shall assist the amputee to interact with his/her prosthesis via the sensory system. Thus, sensory system selection may assist the implementation of the control algorithm of the prosthetic knee and could provide alternative solutions for measurement of the interface pressure inside the stump.

Another challenge nowadays is how to find new methods of measuring the interface pressure for transfemoral amputees. Measuring the interface forces between the socket and the stump could provide information about the socket fabrication in the lower amputation field [21,22]. To date, the interface pressure for transfemoral amputees has not been clearly investigated, due to the shape of the stump that may vary from one amputee to another. Researchers have attempted to predict the amount of forces generated inside the stump of transtibial and transfemoral amputees. One study on interface pressure inside the stump measured it for transtibial amputees using F-socket transducers 9811E (Tekscan, Inc., South Boston, MA, USA) in which the transducers were attached to the posterior, anterior, lateral, and medial compartments of the stump to obtain better insights into the pressure between the stump and socket. The trials were conducted for the amputees during stair ascent and descent, and the study revealed that a high interface pressure exists between the stump and socket with the Seal-In X5 interface system [23]. A Flexforce network sensor made up of five Flexforce elements was used to measure the pressure inside the stump for transfemoral amputees, in which the study reported the amount of forces that can be measured at the x-direction which was about 26 N [24]. Another attempt was performed by using a Fiber Bragg grating (FBG) sensor that was developed to measure the interface pressure between stump and interface socket for transtibial amputees [25], where the range of measurement of the FBG was reported to be about 30 N. The study reported acceptable behavior of the FBG in terms of linear relationship between the shift in the peak wavelength and the applied force. The piezoelectric bimorph can be easily embedded inside the socket to measure the interface pressures at specific regions of the lower limb where high pressure is expected, such as at the posterior, posterior distal, or interior regions [26]. In this paper an investigation of the usage of piezoelectric bimorphs in the field of prosthesis is reported. More specifically, the current approach aims to determine the static and dynamic behavior of the piezoelectric bimorphs in order to utilize them as a sensory system inside a transfemoral amputee's prosthesis socket. Moreover, transient and frequency response analysis were performed to provide information about the response time and the frequency response which would provide useful information during dynamic applications. To validate the piezoelectric bimorph performance in a real situation, an experiment with a single transfemoral amputee subject was conducted while wearing a socket embedded with piezoelectric bimorphs that were placed at different socket sites. The experiment aimed to identify the variation of piezoelectric bimorph performance at different socket sites during the amputee's stride.

## 2. Materials and Methods

A piezoelectric bimorph (T220-A4-503X, Piezo Systems, Inc., Woburn, MA, USA) was selected as the sensory element in this work. Its static and dynamic characteristics were investigated. The piezoelectric bimorph was intended to be utilized as a sensing element inside the socket of transfemoral amputees. Detailed procedures for investigating the static and dynamic characteristics of the piezoelectric bimorph are presented in the following sections.

### 2.1. Piezoelectric Bimorph Characteristics

**Figure 1.**
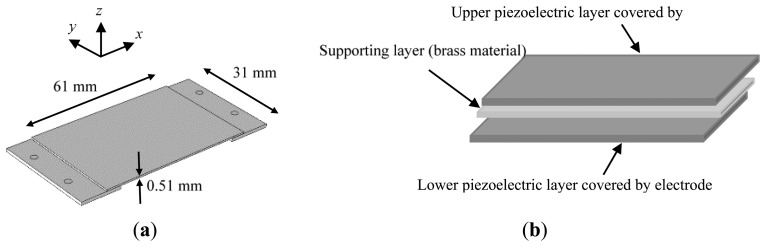
(**a**) Basic dimensions of the piezoelectric bimorph in a simple supported beam configuration; (**b**) Piezoelectric bimorph consists of two layers sandwiched with supporting layer.

In order to assess the overall characteristics of the piezoelectric bimorph, a calibration procedure was conducted to estimate the static and dynamic behavior of the piezoelectric bimorph. The piezoelectric bimorph consists of two layers sandwiched by brass layer as shown in Figure 1.

**Figure 2.**
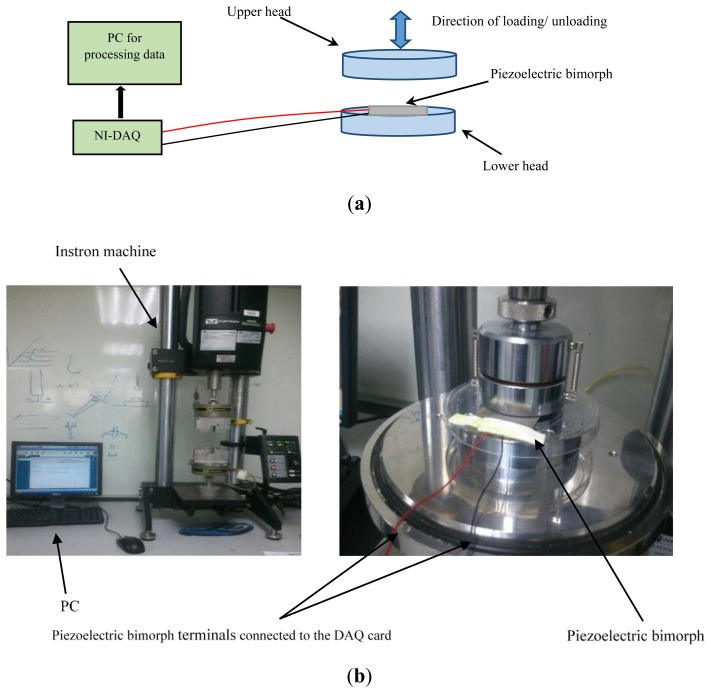
Overall diagram of the sensor calibration. (**a**) Simple schematic illustrating the calibration experimental setup; (**b**) Experimental setup.

A series of input signals were applied to the input of piezoelectric bimorph and its corresponding outputs were recorded. In the current approach, static and dynamic calibrations were performed on the piezoelectric bimorph. Figure 2a shows a simple schematic of the calibration experimental setup. Also, an Instron machine (Instron Worldwide Headquarters^®^, Norwood, MA, USA) was used to perform the calibration of the piezoelectric bimorph as illustrated in Figure 2b. The machine consists of two lower and upper heads, while the bimorph was fixed on the lower head with the force exerted from the upper head. The purpose of the bimorph calibration is to set the static and dynamic behaviour of the bimorph that is used in development and fabrication of the active amputee's socket [21]. The calibration procedure was conducted by applying specific loads to the piezoelectric bimorph in both static and cyclic form to mimic the real situation of pressure dynamics inside the socket.

### 2.2. Calibration Procedure

#### 2.2.1. Static Characteristics

In this section the static characteristics of the piezoelectric bimorph were investigated. To predict the static characteristics, a series of independent-time input values were sent to the piezoelectric bimorph and the output will increase to a level that is proportional to that input. Independent-time values mean that values of inputs do not change with time. The output will remain at that level until the input level is changed. Static characteristics such as sensitivity, hysteresis, range, linearity, and repeatability were referenced in the current work to evaluate the piezoelectric bimorph's response. The benefit of the calibration is to get the characteristics of the sensor, one of the calibration techniques that can provide accurate measurements and collect data in short time is the motion and shape approach [12]. However, one of the limitations of that technique appear in the dynamic measurements as the sensor should be moved with minimum acceleration to make the measurements quasistaic. In addition, the common calibration method of the force sensor is performed by loading input forces on the sensor element. Afterwards the output voltage is recorded [27,28]. The common calibration procedure uses a loading device such as a loading plate, weights, and such a base. Here, the calibration technique that was adopted uses a standard calibration machine in which, a known force value from an Instron (Microtester 5848) strain machine was produced. The corresponding voltage output from the bimorph was recorded simultaneously. The calibration was done in the range of interest, because measurements within the range of interest will assist to enhance the bimorph's sensitivity and resolution. A schematic view of the piezoelectric bimorph that was placed between the machine's heads is shown in Figure 2b. The applied compressive force started at 0 N and increased up to 100 N. The rated deflection and force were recorded *versus* the corresponding output voltage of the piezoelectric bimorph. Data were acquired with both increasing and decreasing loads steps to highlight the hysteresis characteristics. The sensitivity of the piezoelectric bimorph was determined by calculating the slope of the static calibration curve.

#### 2.2.2. Dynamic Characteristics

One of the significant characteristics of the bimorph is the capability to measure different parameters such as force or displacement while the input varies with time. Dynamic characteristics show the behavior of the bimorph during dynamic applications. Each bimorph has the ability to measure static and dynamic movements up to a specific range. Basically, the piezoelectric bimorph was evaluated to predict its behavior when exposed to a family of variable dynamic input waveforms such as a sinusoidal function to obtain the frequency response and a square signal to find out the response time and the damping [29].

The transfer function can be derived to attain a relation between input and output of the piezoelectric bimorph. The piezoelectric bimorph element can be modeled as a simple vibratory system (spring-mass-damper system) [30,31], that presents the analytical dynamic behavior of the piezoelectric. The output voltage *versus* the input force can be provided in terms of damping coefficient and frequency. The dynamic response of the bimorph was described as a second-order system a Laplace form as in Equation (1) [24]:

(1) $$\frac{C\left(s\right)}{R\left(s\right)}=\frac{\omega_{n}^{\text{2}}}{s^{\text{2}}+\text{2}\xi \omega_{n}s+\omega_{n}^{\text{2}}}$$

where *C(s)*, the output of the system, *R(s)*, input to the system, *ω_n_*, natural frequency of the system, *ξ*, Damping of the system.

The behavior of the second order system is described by *ξ* and *ω_n_*; as an assumption damping of ξ = 1 is considered. Therefore, *C(s)* for *R(s)* = *1/s* was expressed as in Equation (2):

(2) $$\frac{V\left(s\right)}{F\left(s\right)}=\frac{\omega_{n}^{\text{2}}}{{\left(s+\omega_{n}\right)}^{\text{2}}s}$$

where *V(s)*, the output voltage, *F(s)*, applied force to the bimorph.

The inverse Laplace transform of Equation (1) may be written in the time domain as in Equation (3):

(3) $$c\left(t\right)=1-e^{-\omega_{n}t}\left(1+\omega_{n}t\right),\;\text{for}\;t\geq 0$$

Equation (3) is essential to characterize the dynamics of the piezoelectric bimorph, that is useful at the overall closed loop system control.

**Figure 3.**
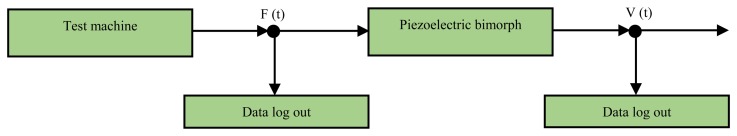
Block diagram shows the procedure of measuring the frequency response of the piezoelectric bimorph.

The dynamic input wave forms were generated by the 5800 series Instron machine, that includes Advanced Cyclic WaveMaker Software^®^, that could generate sine and square waveforms [32]. The response was acquired by a DAQ system (NI USB 9221, National Instruments^®^, Austin, TX, USA) for further processing. The dynamic characteristics namely the frequency response, response time, and damping have been highlighted to evaluate the bimorph behavior [33]. In order to estimate the operating frequencies of the piezoelectric bimorph, a sinusoidal wave was chosen as an input to validate the transient characteristics of the bimorph. Then, the frequency response curves due to the change of the input frequencies were plotted. The diagram to perform the frequency response is shown in Figure 3. The frequency response was tested with different force levels to predict the bandwidth of the bimorph.

Labview software was utilized to process the acquired data from the NI USB 9221 DAQ system. The overall procedure of acquiring the data was established. To obtain the whole set of data during the calibration procedure, an interface was developed using Labview software to save the data for post-processing.

### 2.3. Theoretical Calculation of Loads at the Knee Joint

**Figure 4.**
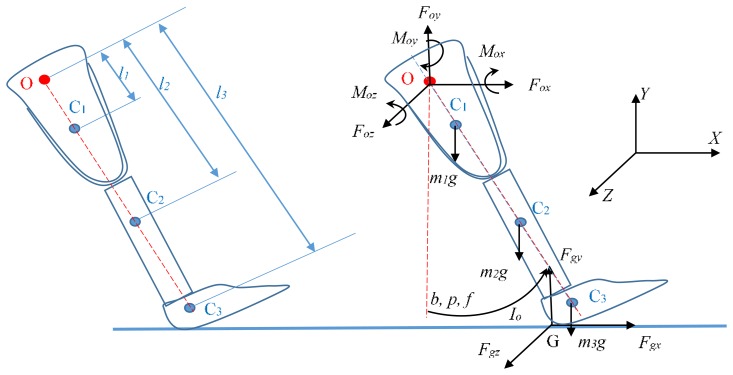
Free body diagram of forces and moments during prosthetic leg heel strike [34].

To measure the interface pressure at the lower limb prosthesis, the mechanical concept of forces and moments were calculated. Basically, forces and moments that are present at a prosthetic device are generated due to the contact with the ground. These forces and moments transferred to interface the amputee. The dynamic analysis is basically based on Newton's second law, with the calculation of the forces and moments [34]. Figure 4 shows the diagram of forces and moments relative to the *x, y, z* axes.

Equation (4) shows the knee rotation by the sum of moments with respect to the origin *O* (Figure 4):

(4) $$M_{oz}-m_{1}g_{1}l_{1}\sin\left(b\right)-m_{2}g_{2}l_{2}\sin\left(b\right)-m_{3}g_{3}l_{3}\sin\left(b\right)+F_{gx}y_{g}+F_{gy}x_{g}=\;I_{o}f$$

where, *b* is the angular displacement on sagittal plane; *m_i_* (*i* = 1, 2, 3) are the stump masses, socket with the tube and prosthetic foot respectively; *l_i_* (*i* = 1, 2, 3) are the distances from the center of mass to the origin *O*. Equation (5) shows the sum of moments *x* regarding *O*:

(5) $$M_{ox}+F_{gz}y_{g}+F_{gy}z_{g}=\;0$$

Equation (6) shows the sum of moments *y* regarding *O*:

(6) $$M_{oy}+F_{gz}x_{g}+F_{gx}z_{g}=0$$

Equations (7)–(9) show the sum of forces on the different coordinated axis (*x, y, z*):

(7) $$F_{ox}+F_{gx}=\left(m_{1}+m_{2}+m_{3}\right)\;\left(\text{rfcos}\left(b\right)‐rp^{2}\text{sin}\left(b\right)\right)$$

(8) $$F_{oy}+F_{gy}‐\left(m_{1}+m_{2}+m_{3}\right)g=\left(m_{1}+m_{2}+m_{3}\right)\;\left(\text{rfsin}\left(b\right)‐rp^{2}\text{cos}\left(b\right)\right)$$

(9) $$F_{oz}+F_{gz}=0$$

where *p* is the angular velocity, *f* is the angular acceleration and *r* is the distance from the origin to the center of mass of the entire model. The interface pressure is described according to the previous equations. According to Equations (7)–(9), the maximum exerted force was 26.1 N. Based on the calculated values of the maximum exerted force, a sensor that can be used to measure the generated pressure was selected. Therefore, an experimental case of using the piezoelectric bimorph to measure the stump/socket pressure for transfemoral amputees were undertaken.

#### Transfemoral Subject Trials

**Figure 5.**
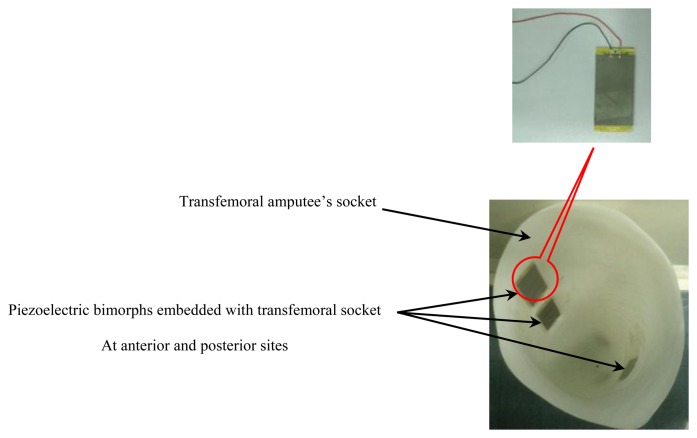
Piezoelectric bimorph sensors embedded to the transfemoral amputee's socket.

To investigate the approach of using a piezoelectric bimorph to detect the stump/socket pressure for a transfemoral amputation subject, an experiment with a single amputee was conducted. The piezoelectric bimorphs were placed in particular socket regions to acquire the maximum amount of pressure from the lower limb that would provide an indication about the gait characteristics. Three piezoelectric bimorphs were embedded to transfemoral amputee's socket as shown in Figure 5.

The session was started by asking a transfemoral amputee subject (age 29 years old, male, 75 kg, height 182 cm) who had been wearing an above knee prosthetic leg for the past 10 years, to perform walking movements at self-selected speed. Piezoelectric bimorphs were inserted in three different locations inside the socket at the anterior proximal, anterior distal, and posterior positions in which the maximum stresses are generated from those sites [22,31,35]. The bimorphs were tethered and the output signals were transmitted via wires to the workstation.

## 3. Results and Discussion

### 3.1. Results and Discussion of the Static Test

**Figure 6.**
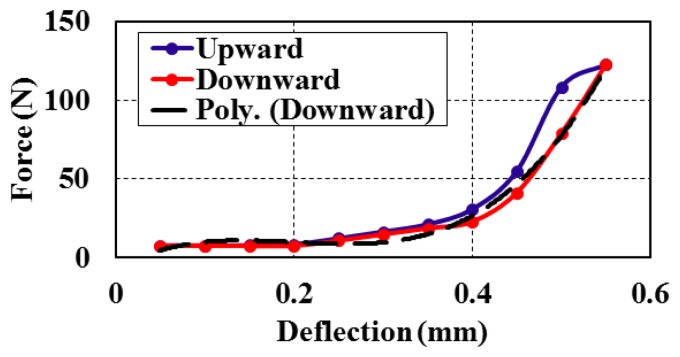
Relation between applied force *versus* the deflection of the piezoelectric bimorph.

**Figure 7.**
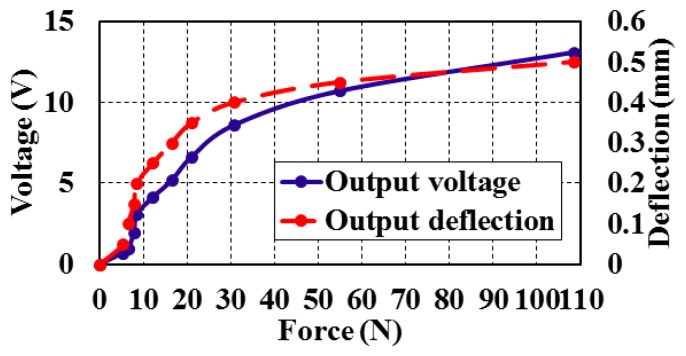
Force *versus* output voltage and deflection.

The static characteristics such as range, linearity, hysteresis, and repeatability were presented to show the bimorph behavior and the operating range at static measurements. Figure 6 shows the output force *versus* the vertical deflection at *z*-direction in reference to Figure 2a. A hysteresis effect was also determined by measuring forces in the upward and downward directions (Figure 6). Figure 7 shows the relation between the input force *versus* the output voltage and the deflection at different values of forces that ranged from 0 to 120 N.

**Figure 8.**
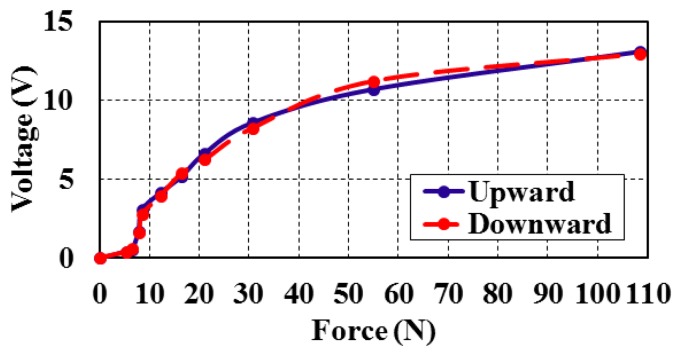
Output voltage from the sensor *versus* the applied force in upward and downward directions.

**Figure 9.**
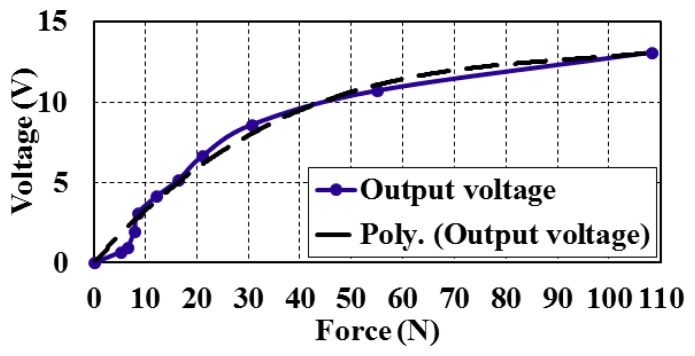
Output voltage of the piezoelectric bimorph *versus* the applied force showing the regression line.

The bimorphs' output voltage and deflection can be calculated against the input force value within the range of measurements (Figure 7). The full scale output (FSO) hysteresis of the piezoelectric bimorph at the applied forces during upscale and downscale was calculated and is shown in Figure 8. The sensitivity and linearization can be figured out by plotting the regression line of the piezoelectric bimorph data as shown in Figure 9. The static validation of the piezoelectric bimorph shows a capacity of measuring forces up to 100 N under static operation conditions (Figure 8).

**Figure 10.**
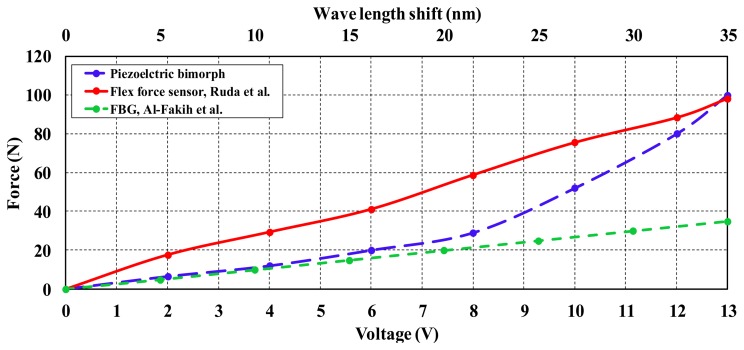
Static force characteristics of piezoelectric bimorph and two different available force sensors.

Further comparison between the adopted piezoelectric bimorph and the other existing sensors would be useful to show the differences in terms of the linearity and the range of measurement. Figure 10 shows a comparison that was conducted between the piezoelectric bimorph, FBG sensor, and Flexforce sensors [24,25]. The FBG sensor can measure force and produce s wave length shift that predicts the amount of force. As shown in Figure 10, FBG exhibits an acceptable linearity along the scale of measurements. However, the range of force was smaller compared to the piezoelectric bimorph and Flexforce units. The piezoelectric bimorph has a range of static force measurements of 0–100 N as shown in Figure 10, which is almost same as the Flexforce that has a range of 0–98 N. While both the piezoelectric bimorph and Flexforce have almost a similar range in terms of static force, the piezoelectric bimorph has better dynamic characteristics in terms of dynamic range and operating bandwidth, 0–77 N and 0–35 Hz, respectively (Table 1). However, The Flexforce has a limitation in the dynamic range when it is placed on curved surfaces, as the effective area is bent and this affects the dynamic response of the device [36].

**Figure 11.**
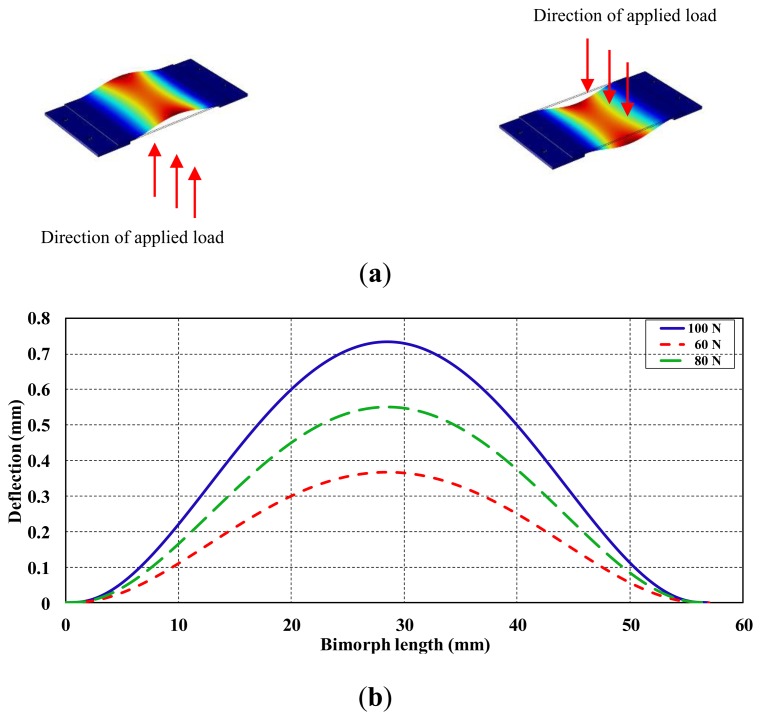
Performance of the piezoelectric bimorph, (**a**) Bimorph deflection when load applied at both faces; (**b**) Piezoelctric bimorph's deflection at different applied loads, simulation performed using Comsol software.

A simulation was conducted using Comsol Software^®^ to illustrate the show the deflection behavior of the bimorph under different applied forces (Figure 11a). The bimorph was deflected according to the amount of load that was applied to the surface area. The forces were applied at 60, 80 and 100 N, respectively, and the bimorph's corresponding deflection along the length was recorded and plotted. The bimorph showed variations of the deflection values along with different loads, with a maximum deflection of 0.73 mm at 100 N applied force. The maximum values of the deflected bimorph occurred at the middle of the bimorphs length as shown in Figure 11b, thus, the current simulation will assist to better understand the behavior of the bimorph when the real interface pressure of the amputee subject is considered. Furthermore, the surface area of the piezoelectric bimorph (0.001085 mm^2^) as shown in Figure 11b provided a wide range of pressure measurement.

### 3.2. Results and Discussion of the Dynamic Tests

The dynamic characteristics basically show the capability of the piezoelectric bimorph under certain dynamic conditions. In this section, the dynamic behavior of the piezoelectric bimorph is represented. The methods adopted to define the piezoelectric bimorph are namely the frequency response, response time, and damping. These methods were adopted to estimate the dynamic behavior of the piezoelectric bimorph in order to determine the functionality of the device in the field of prosthetic knee development.

#### 3.2.1. Frequency Response

**Figure 12.**
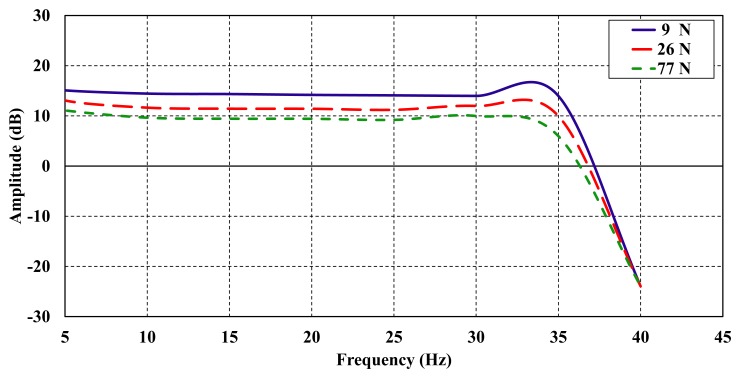
Dynamic response of the piezoelectric bimorph.

The frequency response is a technique to measure the dynamic response of the piezoelectric bimorph. To obtain the frequency response, a harmonic test function (sinusoidal function) was used as an input signal to the piezoelectric bimorph. The sinusoidal input forces selected were 9 N, 26 N and 77 N, to check the functionality of the bimorph under dynamic conditions. The output was monitored and plotted as shown in Figure 12, presented as the frequency response graph of different applied harmonic forces. In addition, it illustrates the operating frequencies and bandwidth of the piezoelectric bimorph. The frequency investigation shows the capability of the bimorph up to 77 N at dynamic region (Figure 12).

#### 3.2.2. Response Time and Damping of the Piezoelectric Bimorph

**Figure 13.**
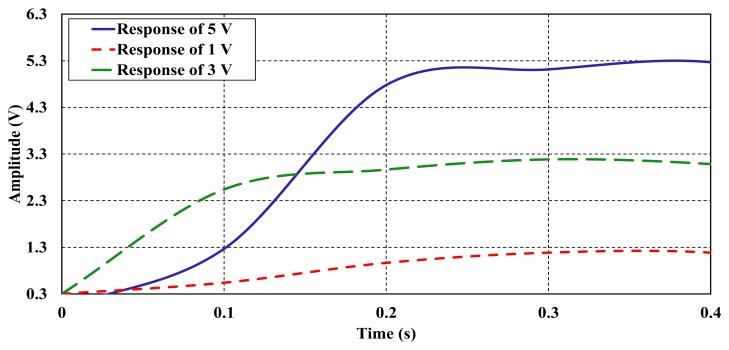
Sample step responses of the piezoelectric bimorph due to different step inputs.

Response time is another means to define the bimorph's dynamic response. Response time is calculated while the bimorph's output reaches a specific percentage of output value when a step change is applied to its input. The step input function is applied to the system to determine the behavior and speed of the system in response to a change in input. Figure 13 shows the transient response of the voltages that were measured at different levels of 1, 3 and 5 V. Particularly, the 5 V response delivered from the piezoelectric bimorph was selected to calculate its response time at 95% and 98%, respectively. The 5 V response produced response times of about 0.22 s and 0.27 s, respectively, which shows an acceptably rapid response for such a level of voltages.

Damping is a sensor's characteristic that defines both how energy from a rapid change in input is dissipated within the bimorph and how it affects the dynamic response characteristics. A critical damping behavior was noticed for the piezoelectric bimorph as can be seen in Figure 13, as it has no overshoot, it is delayed until it reached the final value. An increase in damping in a bimorph may cause the response time and the upper limit of the frequency response to fall. The overall static and dynamic characteristics of the adopted piezoelectric bimorph were obtained and are listed in Table 1.

### 3.3. Results and Discussion of the Case Study

**Figure 14.**
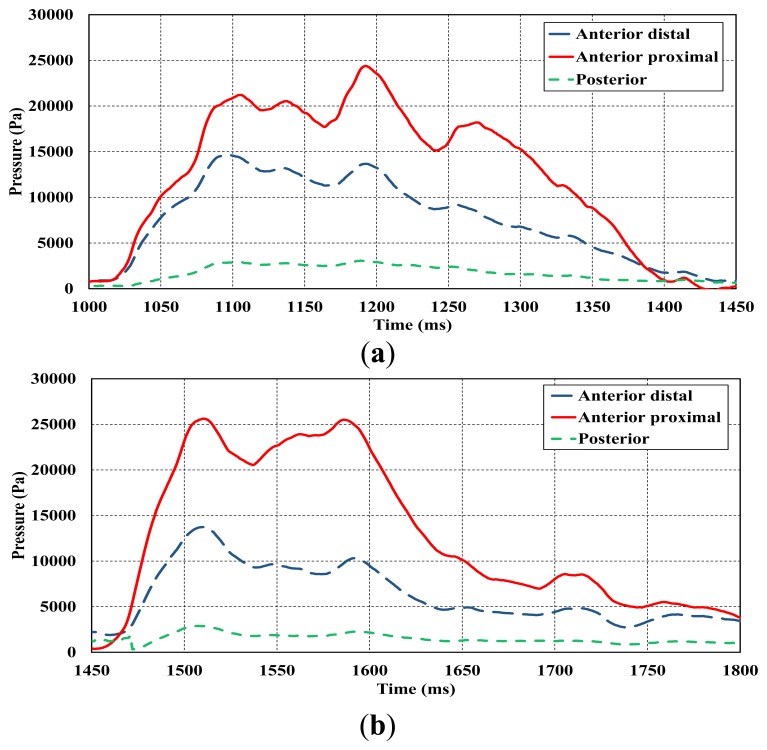

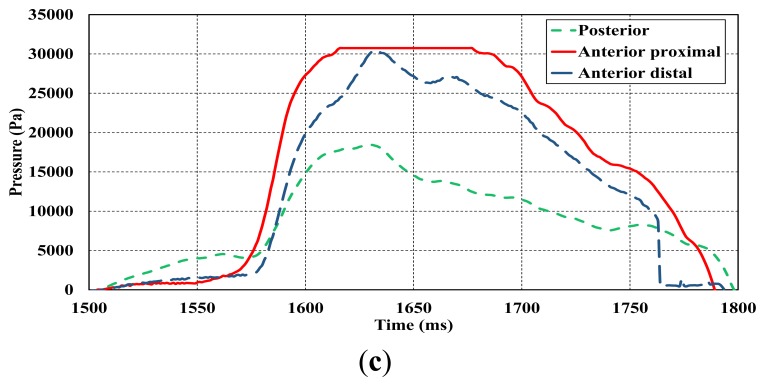
Stump/socket pressure distribution of transfemoral amputee subject during gait, (**a**); (**b**) and (**c**).

The results of the pressure distribution inside the socket during the subject trials were presented in Figure 14. Three tests were performed while the subject was wearing a prosthetic knee device. In each gait test, the pressure at the three locations (anterior, proximal, anterior distal, and posterior) was measured. In Figure 14, the pressure distribution was plotted against a time interval of 450 ms each. The average pressure in the anterior proximal region shows a higher amount of pressure during the tests compared to the anterior distal and posterior regions. This agrees with the results of Dumbleton *et al.* [37], although Dumbleton *et al.*, conducted their study on transtibial amputee subjects who wore the socket for daily use for at least 6 months. Zhang *et al.* [32] considered the pressure interface between the stump and the socket by using finite element analysis. Their research revealed that the distribution of the pressure at the anterior region is higher than the posterior region which emphasised the results of the current study. The maximum pressure that was measured at the anterior region was about 25 kPa as can be seen in Figure 14a,b. However, the piezoelectric showed distortion at the measurement level of 30 kPa during the anterior proximal measurement. Due to the internal properties of the piezoelectric material which affect the hysteresis effect, the coupling effect between the mechanical and electrical parameters became saturated during that level of measurements [14,27].

In this work, the overall characteristics of piezoelectric bimorphs were investigated in order to apply them in lower prosthesis development and interface pressure measurement. A case study was considered to present their capability in that field. More specifically, a preliminary measurement of stump/socket pressure of a transfemoral amputee was considered. Gait socket/stump pressure measurements were conducted because of their significant role in prosthesis research.

## 4. Conclusions

This study was performed to validate the application of piezoelectric bimorph in the prosthetics field. Static and dynamic characteristics of the piezoelectric bimorph were conducted. The dynamic behavior of the bimorph in terms of the response time and bandwidth of operation was investigated. According to the determined characteristics of the piezoelectric bimorph, an assessment of its use as an in-socket sensor was presented. The piezoelectric bimorph sensor was compared to the current Flexforce and FBG sensors in terms of force range and linearity. The piezoelectric bimorph showed similarity to the fFexforce sensor in terms of the static operating range, however the bimorph presented a more suitable dynamic measuring range compared to both the Flexforce and FBG sensors. Furthermore, the current study discussed the usage of the bimorph to measure the interface pressure inside the socket for transfemoral amputee subjects at three different sites. The experiment was conducted with a transfemoral amputee to validate the concept of using the bimorph as a sensing element inside the socket. The results showed that the maximum distribution of the pressure occurs at the anterior region compared to the posterior region. On the other hand at a certain amount of pressure (30 kPa) the signal was truncated due to the saturation of the bimorph's material properties. Thus it can be concluded that the bimorph showed acceptable results for pressure measurements up to 27 kPa and has some limitations for measuring pressures higher than that value. It is recommended to conduct more experiments with subjects of different body weights and pathological considerations to come up with a better understanding of the current approach. Specifically, the measurement of the interface pressure is quite complex due to the combination of normal and shear stress which requires further investigation.

Overall, the preliminary results gathered from the experiments reported in this paper were promising at this stage of research and provided indication about the consistency of the piezoelectric bimorph signals under real measurement conditions. However, more clinical trials utilizing the approach presented in this paper should be performed to validate the capability of the bimorph to measure the shear stresses for both transtibial and transfemoral amputees. Also, more clinical trials with subjects of different weight and level of amputation are recommended. In addition, different activity movements such as sit to stand, slope climbing, and stair ascent/descent could provide further validation of the current concept. Finally, collecting data during clinical experiments could be easier by using a wireless system that facilitates the movement of the subject and provides better handling of the collected data.

## Figures and Tables

**Table 1.** Overall characteristics of piezoelectric bimorph.

**Characteristic**	**Value**
Average sensitivity	0.3 (V/N) of reading
Linearity error	16.8% FSO
Repeatability	1.1% FSO
Static range	0–100 N
Hysteresis	0.4% FSO
Dynamic range	0–77 N
Operating bandwidth	0–35 Hz
Response time at 95% and 98%	0.22 s at 95% and 0.27 s at 98%
Damping	Overdamped
Overall instrument error and uncertainty	1.9%

Where FSO, full-scale operating range.
